# The need for health diplomacy in health security operations

**DOI:** 10.15171/hpp.2019.23

**Published:** 2019-08-06

**Authors:** Vijay Kumar Chattu, Sebastian Kevany

**Affiliations:** ^1^Department of Psychiatry, Faculty of Medicine, University of Toronto, ON, Canada; ^2^Institute of International Relations, The University of the West Indies; St. Augustine, Trinidad and Tobago; ^3^Department of Public Health Research, Global Institute of Public Health, Thiruvananthapuram 695024, India; ^4^Department of HIV/AIDS, University of California, San Francisco, CA, USA

**Keywords:** Health diplomacy, Health security, Global health, Ebola, Health policy, Global health governance

## Abstract

The concept of health security involves the intersection of several fields and disciplines and is an inherently political and sensitive area. It is also a relatively a new field of study and practice which lacks a precise definition - though numerous disciplines and areas like foreign policy, national interests, trade interests, health security, disaster relief, and human rights contribute to the concept. The purpose of this paper is to highlight the need for, health diplomacy in improving health security. For example, it is not unusual for developing country societies to build their health security measures by restricting travel and movement of those emanating from affected areas. When extreme health security measures threaten cordial and cooperative relations between nations, the issue of protection of one country’s population may lead to the risk of international conflict. As the World Health Organization (WHO) stated in 2007 that‘functioning health systems are the bedrock of health security,’ it is crucial that partners with sound financial and technical capacities benefit developing countries through their assistance and sharing information. This paper explores how health diplomacy holds great promise to address the needs of global health security through binding or nonbinding instruments, enforced by global governance mechanisms.

## Introduction

The concept of health security involves the intersection of several fields and disciplines (security studies, foreign policy, global health, and international relations) which do not share a common theoretical approach or academic methodology. Because of this lack of understanding of the concept, there is a risk of disagreements on related policies among developed and developing countries resulting in poor global cooperation. For example, the concept of health security and its application differs between the United Nations bodies namely the United Nations Development Program (UNDP) and the World Health Organization (WHO). The UNDP in its Human Development Report in 1994 identified health security as one of the seven dimensions of human security in its “New Dimensions of Human Security” which led to the linkage of health concerns/ issues to human security.^1^ In contrast, the WHO constitution states that ‘the health of all peoples is fundamental to the attainment of peace and security’^2^ where health contributes to global security rather than securing health itself. The World Health Assembly’s resolution of 2001 ‘Global health security: epidemic alert and response’ linked the concept of health security for the prevention of movement of communicable diseases across national borders which resulted in the revision of the WHO’s international health regulations (IHRs).

## Health security in the contemporary world

Health security is as an inherently political and sensitive area. With any new bilateral or multilateral policies or measures, the risk of damage to relationships between the developed and developing world is threatened. Often, in situations such as the 2014 Ebola crisis, health measures are delivered in a knee-jerk, panicked and self-interested ways rather than with the best interests of global conditions or of the affected country at heart. These type of responses occur naturally, if Darwinist, reaction to disease threats. In the middle ages and as recently as the 20^th^ century, it was not unusual for developing country societies to build their health security measures by restricting travel and movement of those emanating from affected areas.

In the same way, the current danger lies with the positional slide from protectiveness to the employment of health security arguments to discourage unwanted human migration, and therefore globalization. In such cases, it is crucial that — even in emergencies — that health security is tempered by health diplomacy.

When extreme health security measures threaten cordial and cooperative relations between nations, at what point does the priority switch from the protection of one country’s population against (in the worst case scenario) the risk of international conflict? This question is compounded by the often invisible effects on international relations of such interventions, many of which may not come to light until years later after post-intervention assessments and evaluations are completed. Such oversights are compounded when one further considers the multiplex of actors in the health security realm, from the military to epidemiologists to non-governmental organizations. In the current scenario, we have even more actors such as the Global Fund, the United Nations (UN), and local and international ministries of health. This includes the use of domestic and foreign military resources for health care — an evolution in roles and responsibilities that is increasingly important to both strategies and structures of 21^st^-century armed forces.^3^ In the case of the Ebola response in Sierra Leone, the rapid response nature of the military in corralling and controlling the epidemic — including the expedited construction of so-called internment centers -- bore many of the qualities of a counter-insurgency operation against a ballistic threat or human enemy. The WHO stated in 2007 that ‘functioning health systems are the bedrock of health security.’ It is therefore crucial that the partners with sound financial and technical capacities benefit developing countries through their assistance and sharing information by following health security concepts.^4^

## Health diplomacy and its growing importance

Health diplomacy is a relatively a new field of study and practice which lacks a precise definition, though numerous disciplines and areas like foreign policy, national interests, trade interests, health security, disaster relief, and human rights contribute to the concept. The terms health diplomacy, global health diplomacy, and medical diplomacy are often used interchangeably in the current literature. In the health security context, the most appropriate definition is related to the interface between international health assistance and international political relations. It may also be well-defined as a political change agent that meets the dual goals of improving global health while helping repair failures in diplomacy, particularly in conflict areas and resource-poor settings.”^5^

As with any rapid response — particularly those using hard power —, the end can often justify the means. Yet in a rush to contain the epidemic, the presence and philosophy of military operators had to be managed with extreme care. In turn, this highlights the particular importance of global health diplomacy – at both community and national levels – when armed forces become involved in health responses. While the creation of an adequate yet also humanitarian use for defense funding is to be welcomed, institutional cultures of combat, containment, and adversarialism also need to evolve and adapt to a new type of biological rather than human adversaries. The interface of the realms of global public health, international relations, and health security, therefore, demands finesse as well as a force: too easily, soldiers and armies can mistake local communities for the enemy, rather than the virus they are carrying.

In spite of these opportunities for improvement, the military response to Ebola,^6^ for example, can be considered as a proven and dramatic success – based on no more compelling evidence that the absence of a second epidemic in Sierra Leone in recent years. The virus related to H1N1 strains are still active and have a potential of spreading across the globe and in 2017, there were outbreaks that had the potential to spread across the regions.

Apart from these outbreaks, the global community is facing another health security challenge that is the increasing anti-microbial resistance in the pathogens that can infect both humans and animals which again needs effective global governance mechanism to ensure specific standards and frameworks are followed to combat the global spread.^7^ To address these global threats effectively, we need multi-sectoral and multilateral efforts such as the Global Health Security Agenda (GHSA) is an effort by nations, international organizations, and civil society to speed up the progress towards a safe and secure world from infectious disease threats. GHSA is a result of successful health diplomacy efforts primarily to promote global health security as an international priority and ensure the participating countries implement the security frameworks. It aims to spur progress towards implementation of the WHO’s IHRs, the World Organization for Animal Health (OIE) Performance of Veterinary Services (PVS) pathway, and other relevant global health security frameworks.^8^

Globally, it is recognized that the chronic non-communicable diseases (NCDs) also pose a threat to the health security of the nations, regions and now became a global epidemic. The high-level commitment, lengthy persistent negotiations and successful health diplomacy efforts in the Caribbean region over a decade resulted in the Regional Summit declaration “Port of Spain Declaration” in 2007. This declaration gave a comprehensive policy with action points for the prevention of NCDs in the region.^9^ After continuous negotiations through numerous diplomatic rounds, this Summit declaration was deliberated at UN level which finally resulted in the United Nations Political Declaration for the Prevention and Control of NCDs and later to the WHO’s Global Action Plan for the Prevention and Control of NCDs. Another excellent result of global health diplomacy is “The Framework Convention on Tobacco Control” (FCTC) which has global reach by binding all the WHO member states and its negotiation process. These negotiations are one of the first examples of various countries and non-state entities coming together to create a legally binding instrument to govern global health.^10^

## Conclusions

Health diplomacy holds a great promise to address the needs of global health security through its binding or nonbinding instruments enforced by global governance institutions.^11^ GHSA, FCTC, Port of Spain Declaration, United Nations Political Declaration on the Prevention and Control of NCDs and WHO Global Action Plan for the Prevention and Control of NCDs are some excellent examples of successful health diplomacy in the recent years. These global commitments primarily aim to promote global health security as an international priority and ensure the security frameworks are implemented by the states thus emphasizing the need and emerging role of global health diplomacy.

## Ethical approval

The review is part of doctoral research of the first author (VKC) which was approved by the Campus Ethics Committee at The University of the West Indies, Trinidad and Tobago (CEC723/09/18).

## Competing interests

The authors declare that they have no competing interests.

## Funding

The study did not receive any funding.

## Authors’ contributions

Both the authors SK and VKC contributed equally. SK and VKC did the conceptualization, design, initial draft, manuscript revisions and both have approved the final draft.

## Acknowledgments

The authors would like to thank all the researchers and authors of those documents that were referred in the preparation of this manuscript.
